# Epidémie de choléra au Burkina Faso en 2005: aspects épidémiologiques et diagnostiques

**DOI:** 10.4314/pamj.v8i1.71047

**Published:** 2011-01-16

**Authors:** Carole Gilberte Kyelem, Alain Bougouma, Rigobert Sankardia Thiombiano, Ida Adeline Salou-Kagoné, Lassané Sangaré, Ramata Ouédraogo

**Affiliations:** 1 Service de Médecine Interne, Centre Hospitalier Universitaire Sourô Sanou, Bobo-Dioulasso, Burkina Faso; 2 Service d’Hépato-Gastro-Entérologie, Centre Hospitalier Universitaire Yalgado Ouédraogo, Ouagadougou, Burkina Faso; 3 Service des Maladies Infectieuses, Centre Hospitalier Universitaire Yalgado Ouédraogo, Ouagadougou, Burkina Faso; 4 Laboratoires d’Analyses Médicales, Centre Hospitalier Universitaire Yalgado Ouédraogo, Ouagadougou, Burkina Faso; 5 Laboratoires d’Analyses Médicales, Centre Hospitalier Universitaire Charles de Gaulle, Ouagadougou, Burkina Faso

**Keywords:** Epidémie, choléra, Vibrio cholerae, Burkina Faso

## Abstract

**Introduction:**

L’objectif de cette étude était de décrire les aspects épidémiologiques et diagnostiques de l’épidémie de choléra au Burkina Faso en 2005.

**Méthodes:**

Etude rétrospective, d’août à octobre 2005. Elle a concerné dix districts sanitaires du Burkina Faso. A été inclus dans l’étude, tout patient présentant un syndrome cholériforme, admis dans les différentes formations sanitaires dont la coproculture s’est révélée positive à *Vibrio cholerae*.

**Résultats:**

Au cours cette épidémie, 1050 cas de diarrhées cholériformes ont été notifiés par l’ensemble des structures sanitaires du pays. *Vibrio cholerae* a été identifié à l’examen bactériologique des selles de 121 patients (17,2%), constituant notre population d’étude. Les hommes étaient majoritaires (57%). La moyenne d’âge était de 30 ans. Les femmes au foyer (24%) et les sujets non scolarisés (62,8%) représentaient les couches sociales les plus touchées. Les forages ont été la source de boisson de 39,7% des patients 72 heures avant le début de la maladie. Tous les patients ont présenté une diarrhée aqueuse. *Vibrio cholerae*, sérotype Ogawa, responsable de cette épidémie, était résistant au chloramphénicol et au cotrimoxazole dans respectivement 71,7% et 38,3% des cas. Ni le cas index, ni la source initiale de contamination n’ont pu être identifiés. La létalité de notre échantillon était de 3,5%.

**Conclusion:**

Cette épidémie a relancé la question de l’hygiène et mis à nu le problème de ces villes où la croissance démographique galopante est en inadéquation avec le degré d’urbanisation.

## Introduction

Le choléra demeure encore hélas, d’une actualité tristement banale, sévissant sur le mode endémo-épidémique dans plusieurs régions d’Afrique, d’Asie et d’Amérique latine [1,2]. Depuis 1817, sept pandémies ayant toutes comme point de départ l’Asie, ont envahi le monde. L’actuelle pandémie, la septième, intéresse grandement l’Afrique depuis 1970, où elle y est caractérisée par une endémisation de la maladie. Vibrio cholerae y circule sous la forme de ses deux sérotypes majeurs : O1 et O139 [1].

Le Burkina Faso, pays en développement situé au cœur de l’Afrique occidentale dans la boucle du Niger, n’échappe pas à ce fléau. Dès 1971, l’on y enregistrait 1760 cas de choléra dont 501 décès, soit une létalité de 28,5%. En 1973, 1114 cas furent notifiés, dont 241 décès avec une létalité de 21,6%. En 1974, le nombre de cas diminue, de même que la mortalité: 632 cas dont 66 décès et 10,4% de létalité. Puis, cinq grandes épidémies ont été enregistrées en 1991, 1995, 1998, 2001 et 2005 [3]. Depuis lors, aucun cas de choléra n’a été notifié par les services de santé du pays.

Dans le but de mieux appréhender cette maladie et d’y déceler ses spécificités, notre étude se propose, de présenter les aspects épidémiologiques et diagnostiques de l’épidémie de choléra qui a sévi au Burkina Faso en 2005.

## Méthodes

Il s’agissait d’une étude rétrospective, qui a couvert la période d’août à octobre de l’année 2005. Elle a concerné dix districts sanitaires répartis dans cinq régions sanitaires du Burkina Faso. A été inclus dans l’étude, tout patient présentant un syndrome cholériforme, admis dans les différentes formations sanitaires du 12 août au 30 octobre 2005 et dont la coproculture s’est révélée positive à *Vibrio cholerae*. En ont été exclus les patients n’ayant pas bénéficié de coproculture ou dont la coproculture était négative à *Vibrio cholerae*. Les selles ont été prélevées fraiches ou par écouvillonnage rectal. Elles ont été acheminées au laboratoire soit dans des tubes secs ordinaires, ou mieux dans le milieu de transport Cary Blair.

La collecte des données s’est fondée sur les dossiers des malades, les registres des laboratoires de bactériologie du Centre Hospitalier Universitaire Yalgado Ouédraogo de Ouagadougou (CHU-YO), Centre Hospitalier Universitaire Pédiatrique Charles de Gaulle de Ouagadougou (CHUP-CDG), Laboratoire National de Santé Publique (LNSP) et, à partir des fiches de notification et de d’investigation des cas dans les districts concernés. Les variables d’étude étaient: les renseignements démographiques, le niveau d’hygiène, le mode de contamination, les manifestations cliniques, les examens biologiques, la prise en charge thérapeutique, les modalités évolutives. Le traitement des données a été fait à partir du logiciel Epi Info dans sa version française 3.3.2 et les comparaisons statistiques effectuées grâce au test du chi^2^, avec un seuil de signification p ≤ 0,05.

## Résultats

### Données épidémiologiques

Au cours des dix semaines de cette épidémie, 1050 cas de diarrhées cholériformes ont été notifiés par l’ensemble des structures sanitaires du pays, avec une létalité de 1,5%. Sept cent (700) cas (66,6%) ont bénéficié d’investigations épidémiologiques, diagnostiques, de prise en charge thérapeutique et de suivi. *Vibrio cholerae* a été identifié à l’examen bactériologique des selles de 121 patients (17,2%), constituant notre population d’étude. La létalité de notre échantillon était de 3,5%.

**Figure 1:**
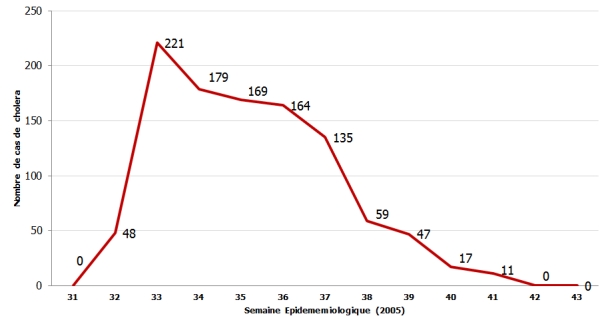
Répartition des cas de choléra en fonction des semaines, au cours de l’épidémie de 2005 au Burkina Faso (Source: Ministère de la Santé, Direction de la Lutte contre la Maladie, 2005)

Le mois d’août a vu le recrutement du plus grand nombre de patients (64%). Il est suivi du mois de septembre au cours duquel 34% des patients ont été enregistrés. La figure 1 montre la répartition de l’ensemble des cas de choléra déclarés dans le pays, en fonction des semaines.

L’âge moyen des patients était de 30 ans, avec des extrêmes de 2 et 74 ans. Les sujets jeunes et actifs de 20 à 39 ans ont représentés un peu plus de la moitié de l’échantillon (51,1%). Les hommes ont représenté 57% des cas contre 43% de femmes, soit un sex-ratio de 1,32. Les femmes au foyer étaient les plus représentées (24% des cas), suivies des travailleurs du secteur tertiaire (19,8% des cas). Venaient ensuite les cultivateurs (17%), les commerçants (17%), les élèves et étudiants (12%) et enfin les salariés (3%). Seuls 25 patients étaient scolarisés (20,6%) dont 20 du niveau primaire et 5 du secondaire.

A l’exception d’un togolais résidant au Burkina depuis quelques années et qui n’avait ni voyagé dans un passé récent, ni reçu d’étranger suspect, tous les patients étaient de nationalité burkinabé.

Les patients de confession musulmane étaient les plus nombreux (66% des cas). Les patients provenaient du milieu urbain dans 83% des cas, et du milieu rural dans 17%. Parmi les citadins, 97% provenaient de la seule ville de Ouagadougou la capitale, dont près de la moitié (45%) du quartier Pissy.

### Modes de contamination

Soixante-douze heures avant le début de la maladie, l’eau de boisson provenait pour 39,7% des forages, 33,1% du robinet et 22,3% des sachets vendus dans la rue, les restaurants et débits de boissons en plein air. De nombreux patients avaient bu l’eau de différentes sources au cours de la même période et certains d’entre eux avaient en plus consommé des boissons locales telles que le zoom-koom (farine de mil cru délayée), le dolo (bière de mil), le bissap (jus de fleurs d’oseille). La majorité des patients (62,8%) ont déclaré avoir mangé à domicile 72 heures auparavant, contre 41,3% qui s’étaient restauré dans la rue et 4,1% dans les restaurants et débits de boissons en plein air.

Les patients ayant eu un comportement à risque dans les 72 heures qui ont précédé le début des signes de la maladie, par contact direct avec un cholérique, une dépouille de cholérique ou toute autre personne suspecte au cours d’un regroupement de population, constituaient 32,2% des cas. Dans 78% des cas, les patients se sont contaminés indirectement par consommation d’aliments, d’eau ou de toute autre boisson souillée. Deux cas de contamination hospitalière ont été notés.

Le genre masculin a été le plus concerné par la contamination indirecte, mais cette association n’est pas significative (p=0,2). De même, les patients musulmans semblaient plus touchés par la contamination directe, mais la différence n’est également pas significative. Il n’existe non plus aucun lien entre la religion et la contamination indirecte. Les femmes au foyer semblaient également plus exposées à la contamination manu portée, mais il n’a été établi aucun lien significatif aussi bien pour la contamination directe que pour celle indirecte. Par contre, il a été noté que le contage en particulier indirect, est très influencé par le niveau d’instruction. Ainsi, les sujets non instruits ont été plus touchés par le mode de contamination indirecte (boissons, aliments souillés) que les sujets scolarisés, la différence étant significative (p=0,02).

### Aspects cliniques

Quatre-vingt-dix pour cent (90%) des patients ont consulté tôt (dans un délai de 6 à 24 heures) après le début de la symptomatologie, tandis que 0,9% n’a consulté qu’au bout de 120 heures (5 jours). Le délai moyen de consultation était de 28 heures.

Tous ont présenté un épisode aigu de diarrhée liquide. Quatre-vingt-six pour cent (86%) des patients ont associé diarrhée et vomissements, tandis que 43,8% ont présenté simultanément diarrhée, vomissements et crampes abdominales. Chez 17 patients, d’autres signes étaient associés: perte de connaissance (3 cas), agitation (12 cas), dyspnée (1 cas) et oligurie (1 cas). L’état général des patients était bon dans 60% des cas, mauvais dans 40% des cas. A l’admission, la conscience était claire chez 89% des patients et altérée chez 11% d’entre eux. Cinquante-trois pour cent (53%) des patients étaient sévèrement déshydratés à l’arrivée, tandis que dans 39% des cas la déshydratation était modérée.

### Aspects bactériologiques

*Vibrio cholerae* a été identifié dans tous les 121 échantillons de selles. Deux sérogroupes ont été identifiés: O1 dans 99,2% des selles et O139 dans 0,8%. Toutes les bactéries O1 étaient du sérotype Ogawa.

L’unique sérogroupe O139 a été isolé chez une patiente de 16 ans, sans emploi, non scolarisée, ayant consommé du riz dans un restaurant les jours précédant sa maladie. Son tableau clinique, de même que le caractère macroscopique de ses selles n’avaient pas de particularités. Par contre, la souche était résistante au cotrimoxazole et au chloramphénicol et sensible aux autres antibiotiques testés. La prise en charge a été identique à celle des autres patients. L’évolution a été favorable, sans aucune complication, le retour à domicile possible après trois jours d’hospitalisation.

En tout, huit antibiotiques ont été testés sur l’ensemble des échantillons, révélant une résistance de la bactérie au chloramphénicol (81%), au cotrimoxazole (57,5%), à l’association amoxicilline + acide clavulanique (20%) et à l’ampicilline (14%). Les vibrions cholériques étaient très sensibles à la ceftriaxone (99%), la ciprofloxacine (99%), l’acide nalidixique (98%) et la doxycycline (92%).

## Discussion

Sur les 1050 cas déclarés, notre étude n’a concerné que 121 patients, soit seulement 17% des cas. L’insuffisance du plateau technique de nos structures sanitaires (insuffisance de réactifs, de milieux d’enrichissement, de milieux de transport) a conduit les formations sanitaires à n’effectuer des prélèvements qu’aux "premiers arrivants" pour confirmation du diagnostic, les autres patients ne bénéficiant plus de prélèvement systématique. Ceci a considérablement réduit notre population d’étude, nous contraignant à nous limiter à la partie visible de l’iceberg.

### Données épidémiologiques

Après vingt ans d’accalmie (1971–1991), le Burkina Faso assiste à une résurgence du vibrion cholérique. La létalité très élevée au départ, s’est réduite pour se stabiliser depuis les dernières épidémies à des taux inférieurs à 2% [4], toujours supérieurs à celui de 1% admis par l’Organisation Mondiale de la Santé [5].

Le taux de létalité de notre échantillon (3,5%), supérieur à celui déclaré officiellement pour l’ensemble du pays (1,5%), révèle une fois de plus les différences de statistiques entre les petites et les grandes séries. Ce taux très élevé, pourrait être dû au fait que nos patients aient été recrutés pour la plupart dans les grands centres de références où les dossiers étaient les plus exploitables mais où il y avait également les malades les plus sévèrement atteints et évacués des centres périphériques pour des complications diverses.

Le début de l’épidémie de 2005 remonte au 12 août, par la notification du premier cas de choléra par le Centre Hospitalier Universitaire Yalgado Ouédraogo (CHU-YO) de la région sanitaire du Centre. Puis, elle s’est étendue en tache d’huile aux régions du Centre, Centre est, Centre ouest, Centre sud. L’épidémie qui a atteint son pic dès la deuxième semaine, s’est poursuivie jusqu’au mois d’octobre, le dernier cas ayant été notifié le 30 octobre 2005 [3]. Sa survenue en pleine saison des pluies corrobore les données de la littérature, qui situe les épidémies de choléra dans les zones sèches pendant la période hivernale ou de fortes précipitations [6-8].

Malgré toutes les recherches effectuées, ni le cas index, ni la source de l’éclosion de l’épidémie de 2005 au Burkina Faso, n’ont été retrouvés.

La tranche d’âge de 20 à 39 ans, la plus active professionnellement, a été la plus touchée (51%), résultats comparables à ceux de Compaoré à Tenkodogo (Burkina Faso) en 2001 [9] et de Jaureguiberry à Madagascar [10]. Nos résultats sont toutefois différents de ceux de Morillon à Djibouti [11], qui rapportait que les enfants de 0 à 5 ans étaient les plus touchés. De même, la prédominance masculine retrouvée dans notre étude (57%), avait été signalée par Tanon en Côte d’Ivoire qui notait 53% d’hommes [12].

Le choléra est aussi considéré comme une maladie de l’ignorance : plus le niveau d’instruction est bas, plus l’on ignore les règles élémentaires d’hygiène et plus l’on est exposé à la maladie et inversement. Dans notre étude, le niveau d’instruction a une influence sur le mode de transmission, les non instruits ont été les plus vulnérables à la maladie (68,2% des cas).

Notre étude comme celle de Compaoré [9], révèle une forte proportion mais non statistiquement significative de patients musulmans. Serait-elle favorisée par certaines pratiques courantes comme l’usage commun de la bouilloire à multiples fins (ablutions, toilette…) dans une famille de surcroît nombreuse comme il en existe beaucoup en Afrique (pas seulement chez les musulmans) ? De même dans nos familles traditionnelles, toutes confessions religieuses confondues, il n’est pas rare que plusieurs personnes boivent dans le même récipient à la suite les uns des autres, mangent dans la même assiette, ou utilisent la même eau pour se laver les mains. Ces pratiques culturelles peuvent et gagneraient à être améliorées, dans le but de réduire l’incidence des maladies hydriques liées au péril fécal.

Quatre-vingt-trois pour cent (83%) des patients provenaient du milieu urbain et parmi ces citadins, 97% résidaient dans la ville de Ouagadougou dont 45% dans le seul secteur 17 (quartier Pissy, à la périphérie ouest de la ville). Les deux centres de traitement qui ont accueilli le plus de malades étaient le Centre Médical de Pissy et le CHU-YO, tous deux appartenant au district sanitaire de Pissy. Konaté au Burkina Faso avait observé déjà en 1984, que 80% de ses malades provenaient de zones d’habitations spontanées de Ouagadougou et des quartiers à forte concentration humaine [13]. Selon l’annuaire 2004 de la Direction des Etudes et de la Planification, pour toute la région du Centre, le district sanitaire de Pissy est le plus peuplé avec plus de 500 000 habitants, suivi du district sanitaire du secteur 30 qui lui, compte environ 300 000 habitants [5]. Pissy, à l’image des autres quartiers périphériques de Ouagadougou, est caractérisé (pour une partie importante de sa population) par la promiscuité, le manque de latrines et de caniveaux, l’insuffisance d’accès à l’eau potable et l’insalubrité.

### Modes de contamination

Les deux cas de contamination hospitalière ont retenus notre attention. Dans nos contrées, rendre visite à un parent, ami ou voisin malade, est culturel, à caractère quasi obligatoire. Déjà en 1984, Konaté notait qu’ « il était impossible durant toute l’épidémie de limiter le nombre des accompagnants à un. Un autre problème est celui des visiteurs qui, malgré le cordon sanitaire formé par la police, franchissaient frauduleusement les haies pour satisfaire leur conscience en allant souhaiter une bonne guérison au voisin malade » [13]. Plus de deux décennies après, nos populations ont gardé les mêmes mentalités, qui changent difficilement. Il a été également constaté que les accompagnants de malades continuent de négliger (par ignorance, défiance ou manque de moyens ?) les mesures préventives préconisées comme la désinfection systématique ou l’incinération de tout objet ou vêtement ayant été en contact direct avec les malades. Cependant, des efforts renouvelés de sensibilisation et l’adhésion progressive des populations, peuvent amener un véritable changement de comportements. En témoigne l’expérience du Sénégal où Ndour [14] ne notait aucun cas de contamination hospitalière en 2004, comme c’était le cas dans son pays lors des épidémies antérieures, témoignant d’une bonne observation des mesures préventives.

### Aspects cliniques

Au cours de l’épidémie de choléra de l’année 2005, la symptomatologie clinique n’a pas différé de celles des années antérieures. Diarrhée faite de selles liquides, vomissements, crampes abdominales, déshydratation plus ou moins sévère, se sont associés à des degrés variables [9,12]. La conscience reste longtemps conservée et, malgré un délai moyen de consultation de 28 heures dans notre série, plus de la moitié de nos patients bien que présentant une déshydratation sévère, avaient une conscience claire à l’arrivée.

### Données bactériologiques

Au plan bactériologique, tous les 121 échantillons de selles analysés ont révélé la présence de Vibrio cholerae. Les sérogroupes O1 et O139 ont été identifiés. Comme partout ailleurs en Afrique les vibrions O1 étaient du sérotype Ogawa [7,12].

Les résultats d’antibiogramme révélaient pour toutes les souches testées une bonne sensibilité aux céphalosporines de 3ème génération, les fluoroquinolones et la doxycycline, une résistance importante au cotrimoxazole et au chloramphénicol et une résistance moindre aux béta lactamines. Morillon à Djibouti en 1994 [11] et Ndour au Sénégal en 2004 [14], faisaient les mêmes constats. Par contre, Dray et collaborateurs, toujours à Djibouti mais en 2001 [6], notaient une sensibilité à 100% à l’ofloxacine, mais une résistance plus ou moins importante aux autres molécules testées: 92% à l’ampicilline, 100% à l’érythromycine, 56% à la tétracycline, 20% à la doxycycline, 91% au triméthoprime, 86,4% aux sulfamides, 88% au cotrimoxazole, 92% au composé O129, 81,8% aux furanes, et 88% au chloramphénicol.

L’importance de l’antibiogramme devant toute épidémie de choléra n’est plus à démontrer et s’impose dans le but de détecter précocement les formes de multi résistance et d’adapter au mieux la thérapeutique.

## Conclusion

L’épidémie de 2005 a relancé la question de l’hygiène et mis à nu le problème de ces villes où la croissance démographique galopante est en inadéquation avec le degré d’urbanisation. Elle a également été très meurtrière, en témoigne le taux de létalité de 3,5% enregistré dans notre étude, largement au-dessus des 1% admis par l’OMS. Malgré des efforts appréciables consentis par tous les acteurs ayant intervenu dans la riposte contre cette maladie (autorités politiques, sanitaires, agents de santé, communauté) et permis de circonscrire l’épidémie en dix semaines, des insuffisances ont été constatées. Certes, la pauvreté et l’ignorance de nos populations sont d’une importance majeure dans le déterminisme et la propagation du choléra et de bien d’autres maladies à potentiel épidémique, mais justifient-elles à elles seules leur persistance et leur sévérité? Une analyse des mécanismes de prévention et des stratégies de riposte aux épidémies déclarées nous permettrait peut-être, de répondre à cette question et d’améliorer la prise en charge de ces maladies à potentiel épidémique.

## Conflit d’intérêts

Les auteurs ne déclarent aucun conflit d’intérêts

## Contribution des auteurs

Carole Gilberte Kyelem: rédaction du document, Alain Bougouma: rédaction du document, Rigobert Thiombiano: collecte, analyse des données, Ida Adeline Salou-Kagoné: collecte, analyse des données, Lassané Sangaré: analyse bactériologique des échantillons, Ramata Ouédraogo: analyse bactériologique des échantillons. Tous les auteurs ont lu et approuvé la version finale du manuscrit.
